# The Influence Mechanism of Reputation Information on the Formation of Safety Trust in Chinese Infant Milk Powder

**DOI:** 10.3390/healthcare8020138

**Published:** 2020-05-20

**Authors:** Yanan Cao, Cuixia Li

**Affiliations:** 1 College of Business, Hebei University of Economics and Business, Shijiazhuang 050061, China; 2 Research Center for Modern Business Service Industry, Hebei University of Economics and Business, Shijiazhuang 050061, China; 3 College of Economics and Management, Northeast Agricultural University, Harbin 150028, China; licuixia@neau.edu.cn

**Keywords:** infant milk powder, safety trust, reputation information transmission, consumer characteristics, psychological perception

## Abstract

Infant milk powder has always been one of the food categories most sensitive to safety reputation information. The safety reputation of Chinese infant milk powder has been seriously damaged due to the occurrence of safety accidents and the resulting consumers’ still-unrestored confidence is an important factor which restricts the dairy industry revitalization. Therefore, this paper analyzes the impact of reputation information on the formation of safety trust in Chinese infant milk powder, taking reputation information transmission as the starting point and consumer psychological perception as the researching perspective. A questionnaire survey was conducted and 685 valid questionnaires were collected. The structural equation model is adopted to verify the theoretical model and corresponding research hypothesis that reputation information affects the safety trust of Chinese infant milk powder. The reputation information transmitted between relatives and friends has a stronger effect on the formation of safety trust in Chinese infant milk powder than the media. The degree of media pursuit of “news effect” and negative word-of-mouth have a significant negative impact on the formation of safety trust in Chinese infant milk powder, while reputation quality, positive word-of-mouth and relationship strength have a significant positive impact on that. The quality of word-of-mouth perceived by consumers from highly involvement group, rational group, urban group and high-educated group has a stronger influence on the formation of safety trust. The degree of media pursuit of “news effect”, positive word-of-mouth, negative word-of-mouth and relationship strength perceived by consumers from low involvement group, emotional group, rural group and low-educated group have stronger influence on the formation of safety trust.

## 1. Introduction

After a series of milk powder safety accidents such as the melamine incident in 2008, information on the quality and safety issues of Chinese milk powder has been widespread. There are different opinions. The negative reputation information of unsafe Chinese infant milk powder (CIMP) causes consumers to have a higher risk perception of Chinese infant milk powder, which seriously affects consumers’ trust [1], and the influence degree is much higher than that of positive reputation information. Many consumers have fallen into the cognitive misconception that all CIMP is unsafe, thus causing rejection of qualified Chinese infant milk powder [2]. In order to change this situation, the Chinese government has strengthened safety supervision on milk sources, factories, production and other sources, intensified dairy industry standards and norms, improved inspection and testing levels, and successively issued more than 30 policies and norms. Enterprises build their own milk source bases, improve their technology and equipment, and open factories, etc. In February 2017, the government, enterprises and trade associations jointly launched “five major actions to revitalize the dairy industry”. In December 2018, “Several Opinions on Further Promoting the Revitalization of Dairy Industry” was issued, proposing to improve the quality and reputation of Chinese infant formula milk powder and to enhance consumer confidence. In May 2019, the “Action Plan for Improvement of Domestic Infant Formula Milk Powder” was issued, further emphasizing the importance of reputation information of infant formula milk powder to enhance consumer confidence and milk industry development, and proposing to improve the safety report and information release system regarding the quality of infant formula milk powder and to strengthen public opinion of propaganda and guidance. Through the continuous efforts of all circles in China, the quality and safety level of CIMP has been significantly improved. According to the announce by the Chinese State Administration of Market Regulation, the qualified rate of sampling inspection on infant formula milk powder in 2019 was 99.79% [3]. However, according to the statistics of the General Administration of Customs of China, the domestic import of infant formula milk powder totaled 345,500 tons in 2019, up 6.5% year on year, accounting for about 8 times of the import in 2008 [4]. Therefore, it can be concluded that the most critical issue in the revitalization of China’s dairy industry is not product safety, but to accelerate the formation of consumer trust [5].However, even if the Chinese government, enterprises and all sectors of society have carried out a series of measures to solve the safety problem of CIMP, why has the negative reputation information of unsafe CIMP not released its impact on consumers? This is a significant issue that needs to be solved urgently in the process of realizing the overall revitalization of the Chinese dairy industry.

The occurrence of a series of milk powder safety accidents such as the melamine incident, has aroused concern and discussion between scholars. The discussion not only includes safety production technology, nutrition and other processing technology fields of infant milk powder [6,7,8], but also includes the quality and safety supervision, industry development, purchasing behavior, and other areas of the economic management in the previous studies [9,10,11,12]. In addition to technology in the field of research, to explore the infant milk powder safety regulation, industry development and purchase behavior, the studies are inseparable from the question that the consumers hold a negative attitude towards CIMP, and the dairy industry, especially infant milk powder industry in our country faces with the serious crisis of confidence [5,13]. So, what factors affect the formation of safety trust in CIMP? Through sorting out the previous studies, it’s found that the studies focused on the discussion of the influencing factors of consumers’ trust in food safety mostly [14,15,16]. The scholars found that the negative reputation information of food safety is an important reason affecting the trust in food safety [13,17,18]. At the beginning of the melamine incident, the trust of Chinese consumers is not completely lost. However, consumers’ trust are damaged and the formation of consumers’ trust is hampered due to the repeated transmission of negative reputation information for a long time after a series of safety incidents gradually [5,13]. So how is the formation of safety trust in CIMP affected by such negative reputational information? Scholars have not explored this issue in depth. Based on this, the paper analyzes the impact of reputation information on the formation of safety trust in CIMP, taking reputation information transmission as the starting point and consumer psychological perception as the researching perspective.

## 2. Theoretical Basis and Research Assumptions

After the occurrence of food safety accidents, consumers may receive a large amount of negative information released by various parties from different channels, and thus overestimating the possibility and severity of potential safety problems [19,20]. Therefore, the formation of consumer trust is affected [5,21,22]. According to the theory of information economics, reputation mechanism is a basic tool to solve food safety information asymmetry. In fact, it is a kind of public opinion with strong informational function [23,24,25]. Consumers can use reputation information to identify the behavioral attributes of CIMP suppliers and infer whether the milk powder they provide is safe or not. And according to the consumer decision-making process theory, it is found that the safety trust in infant milk powder is caused by some kind of stimulus (information), which is received and processed by consumers in the form of information and formed under the regulation of consumer characteristics [26]. Therefore, this paper holds that the key to explore the influence mechanism of reputation information on the safety trust in CIMP lies in the determination of the source, the transmission path and the influence mode of the reputation information, and the characteristics and the influence modes of consumers.

### 2.1. Source Determination and Transmission Path Analysis of Information of the Reputation of Infant Milk Powder

The safety reputation of CIMP refers to the general evaluation of the social public of the safety of infant milk powder. In this paper the social public mainly refers to the media and other consumers such as relatives and friends. The media refers to traditional media such as TV, radio, newspaper and magazine, as well as new media such as WeChat official account, which release the safety reputation information of CIMP to consumers, the word-of-mouth communication of relatives, friends and other consumers refers to the information about the safety reputation of infant milk powder transmitted to consumers by relatives, friends and other consumers through face-to-face conversations, WeChat and other communication tools as well as online communication platforms. The essence of the media is the intermediary of information transmission. The safety of infant milk powder is a matter of great concern to all circles of society, and its reputation information is the focus of media reports. Several Opinions on Further Promoting the Revitalization of Dairy Industry, issued by nine governmental departments, clearly point out that they support vigorous advocacy of achievements made in the development of China’s dairy industry through mainstream media and new media, so as to establish a good image for it and improve consumers’ trust in it. The “Food Safety Law of China” points out that the news media should promote public service announcement on food safety laws, regulations, standards and knowledge, and play a role of public opinion supervision on food safety violations. The media mainly transmit the safety reputation information of infant milk powder to consumers through market supervision and public opinion supervision. New media such as the internet and mobile phones have become the main tools for consumers to obtain reputation information. Relatives, friends and other consumer groups are closely related to consumers and have a relatively stable trust relationship, which is an important information source for consumers to obtain safety reputation information of infant milk powder. Generally speaking, consumers believe that the health and nutrition information provided by health experts is suspicious, while the information provided by family members and friends is mostly reliable and is the main source of information [14,27,28]. Therefore, food safety information such as infant milk powder spread among relatives, friends and other acquaintances affects the decision-making of most consumers [29,30]. When consumers have the demand to buy infant milk powder, most of them would get word-of-mouth information of an enterprise or brand of infant milk powder from their relatives and friends. The main reason is that word-of-mouth communication is usually one-to-one and relatives and friends can choose appropriate communication content and introduction methods according to consumers’ needs. Compared with the marketing activities of enterprises such as advertising or promotional recommendation, the word-of-mouth communication among relatives and friends is easier to win the attention and trust of consumers.

### 2.2. Analysis of the Influence Mode of Reputation Information on Infant Milk Powder

**Figure 1:**
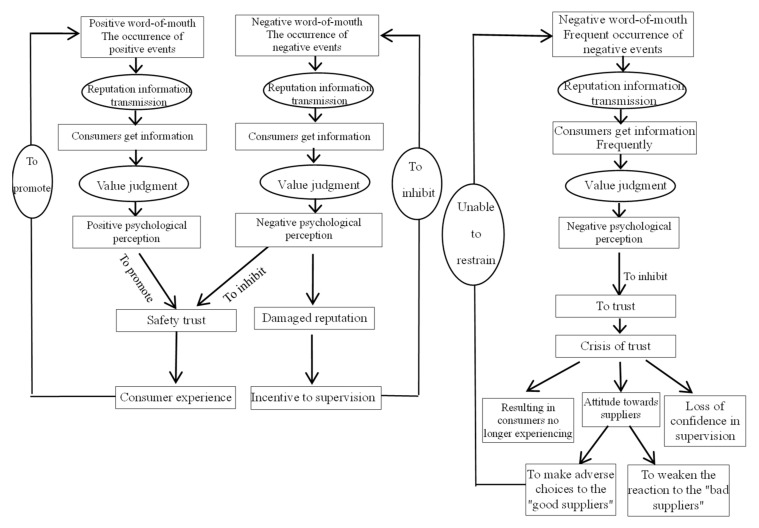
The influence process of reputation information on the formation of safety trust in infant milk powder.

Whether reputation information can be effectively transmitted depends on whether consumers can accept and recognize its intrinsic value after obtaining it [31,32]. What really plays a role is not the information itself, but the psychological perception formed by consumers after obtaining the information, which is also an important variable to explore the “consumer black box” in the consumer decision-making process theory. The positive or negative evaluation through which media and relatives and friends convey reputation information of the safety reputation of CIMP is positive word-of-mouth or negative word-of-mouth. The research on word-of-mouth can be traced back to Arndt, who pointed out that negative word-of-mouth has greater influence on consumers [33]. Positive word-of-mouth will promote the formation of safety trust in CIMP. The media and relatives and friends pass on positive word-of-mouth of the safety of infant milk powder to consumers. After the measure by their own characteristics, consumers make value judgments on positive word-of-mouth and produce certain positive psychological perception. This will promote the formation of safety trust in CIMP, stimulate the consumer experience process, and also play a positive incentive role for various interest bodies to ensure the safety of milk powder. Negative word-of-mouth will inhibit the formation of safety trust in CIMP. The media and relatives and friends pass on negative word-of-mouth of the safety of infant milk powder to consumers. After the measure by their own characteristics, consumers make value judgments on negative word-of-mouth and produce certain negative psychological perception. This will inhibit the formation of safety trust in CIMP, damage the reputation of interest bodies and inhibit the occurrence of negative events. In this occasion, the reputation mechanism is effective. However, if the negative accidents of CIMP occur frequently, the effectiveness of reputation mechanism will be damaged. If consumers often get negative information about the safety of CIMP, they will have negative psychological perception, which will inhibit the formation of trust and generate trust crisis. This kind of phenomenon will cause consumers to be unwilling to take risks to buy CIMP, and to reject all of the CIMP brands and buy imported milk powder in succession. They will make adverse choices to the “good guys” and weaken their reaction to the “bad guys” among the CIMP suppliers. This will lead to bad suppliers driving out good ones, loss of confidence in supervision, etc., and will not restrain the frequent occurrence of negative events [34] (Figure 1). Now, the key point is to find out what kind of psychological perception consumers have from the reputation information of infant milk powder. However, few scholars have studied this kind of psychological perception variable. This research can help all interest bodies in infant milk powder understand the trust decision information preferred by consumers, alleviate the problem of asymmetric reputation information in infant milk powder market, and improve the efficiency and effect of consumer trust decision-making.

First, the influence mode of reputation information transmitted by the media. After the media transmit the safety reputation information of infant milk powder to consumers through market supervision and public service announcement, the safety information plays a role through consumers’ psychological perception, which makes consumers have a certain perception of the degree of media pursuit of news effect and the public service announcement effect of the media and then promotes the formation of safety trust of infant milk powder. The extent to which the media pursue “news effect”. Due to the pursuit of economic benefits, especially some “we media” sometimes repeatedly spread or exaggerate Chinese milk powder safety accidents in order to gain consumers’ attention [35]. Negative reputation will cause social panic and cannot play the role of market supervision [36,37]. At the same time, it will also lead to a decline in consumer trust in government supervision [38], damage the whole milk powder industry and affect the formation of safety trust in infant milk powder. The media publicity effect of public service announcement. The continuous improvement of the media, especially the wide and fast spread of information by new media, has promoted the development of Chinese dairy public service announcement. New media such as the mainstream media and WeChat Official Account and other public service announcement content on milk powder safety laws and regulations, milk powder safety standards and knowledge have greatly affected consumers’ safety trust in infant milk powder. To this end, this paper puts forward the following assumptions:

**Hypothesis** **(H1).** *The media have a significant effect on the formation of safety trust in CIMP.*

**Hypothesis** **(H1a).** *The degree of media pursuit of “news effect” has a significant negative impact on the formation of safety trust in CIMP.*

**Hypothesis** **(H1b).** *The media publicity effect has a significant positive impact on the formation of safety trust in CIMP.*

Second, the influence mode of reputation information transmitted by relatives, friends and other consumers. After relatives, friends and other consumers transmit the safety information of infant milk powder to consumers through word-of-mouth communication behavior, the safety information plays a role through consumers’ psychological perception, which makes consumers have a certain perception of word-of-mouth quality, word-of-mouth type, relationship strength and so on, and then promotes the formation of infant milk powder safety trust. *Word-of-mouth quality*. Consumers’ perception of the quality of word-of-mouth transmitted by relatives and friends is extremely crucial. High-quality reputation information should include detailed and comprehensive introduction of the characteristics or eating experience of infant milk powder by the transmitter, and the information content is well-founded. This makes it easier for consumers to engender a sense of acceptance and promotes the formation of safety trust in infant milk powder. The low-quality reputation information is only a simple recommendation by the transmitter, and it is difficult for consumers to form a sense of acceptance due to the few comments, unclear statements and no reasons [36,39,40]. *Types of word-of-mouth*. Types of word-of-mouth include positive word-of-mouth and negative word-of-mouth. Many scholars believe that negative word-of-mouth plays a greater role than positive word-of-mouth in product sales [33,41], especially for foods such as infant milk powder, of which consumers are highly sensitive to the safety. Consumers’ perception of negative word-of-mouth intensity is more likely to attract consumers’ attention, which will greatly weaken consumers’ trust in the safety of infant milk powder. *Relationship strength*. In the process of building trust, the relationship between the trustor and the trusted person is an important influencing factor. For domestic consumers, the most trusted groups are, in turn, relatives with blood ties, colleagues and friends with business ties, etc. Infant milk powder is one of the most sensitive food categories for consumers. Consumers often seek advice from people with close relationships before purchasing. The closer the relationship is, the greater the impact of reputation information spread by word-of-mouth on the formation of safety trust in infant milk powder [42]. To this end, this paper puts forward the following assumptions:

**Hypothesis** **(H2).** *Relatives, friends and other consumers have a significant positive impact on the formation of safety trust in CIMP.*

**Hypothesis** **(H2a).** *Word-of-mouth quality has a significant positive effect on the formation of safety trust in CIMP.*

**Hypothesis** **(H2b).** *Positive word-of-mouth has a significant positive effect on the formation of safety trust in CIMP.*

**Hypothesis** **(H2c).** *Negative word-of-mouth has a significant negative impact on the formation of safety trust in CIMP.*

**Hypothesis** **(H2d).** *Relationship intensity has a significant positive effect on the formation of safety trust in CIMP.*

### 2.3. Analysis of the Regulatory Effect of Consumer Characteristics on the Formation of Safety Trust in CIMP

Consumer characteristics play a regulating role in the formation process of information influencing trust, which is of great concern to many researchers. According to the Stimulus-Response (S-R) model (JB Watson, 1913), external information stimulus needs to be processed in the consumer black box to ultimately affect consumer decision-making behavior. Mehrabian and Russell further explored the consumer black box through the S-O-R model, confirming that consumer characteristics play a regulating role between information stimulation and consumer response. Existing research materials mainly explore the influence of demographic characteristics of consumers such as age, gender, region and income on consumer trust [15,16,43,44,45], whereas the formation of infant milk powder safety trust is actually a process of understanding the consumer black box, that is, the psychological process of consumers acquiring and processing information. Therefore, this part mainly discusses the psychological characteristics of consumers in acquiring and processing information and the moderating effect of consumer demographic characteristics on the formation of safety trust in infant milk powder. Consumer involvement, emotion (sensibility) system and cognition (rationality) system are selected as psychological characteristics indicators of consumer processing information for analysis, and residential area and level of education are selected as demographic characteristics of consumers for analysis.

In the process of interaction between consumers and the public, the public transmits reputation information to consumers, which has a certain influence on the formation of safety trust. *Consumer involvement*. As consumers with high involvement have searched for detailed information on the reputation of infant milk powder and have paid a certain amount of energy and emotion, their minds have made a certain evaluation on the safety of infant milk powder. And as more and more reputation information is collected, they will seriously consider and compare the reputation information [46,47], so the reputation information of milk powder delivered by the public to consumers has relatively less influence on them. While the consumer’s involvement degree is low, public word-of-mouth information will greatly affect the formation of trust [48]. Therefore, consumer involvement plays a regulating role in the formation process of reputation information influencing the safety trust in CIMP. *Emotional and cognitive system*. The emotional system often includes more emotions and feelings, while the cognitive system is often a rational examination of the credibility of others [49,50,51]. In the process of forming safety trust in CIMP, emotional consumers are more likely to be emotional and pay more attention to the reputation of close relatives and friends and the information in the media. While rational consumers pay more attention to the quality of word-of-mouth and conduct an in-depth analysis on the safety information of CIMP transmitted by the media, thus being relatively less affected. Therefore, emotional system and cognitive system play a regulating role in the formation process of reputation information influencing the safety trust in CIMP*. Consumers’ Residential Areas (urban or rural)*. Urban consumers pay more attention to the word-of-mouth quality of relatives and friends during the formation of safety trust in CIMP. Moreover, they have more contact with the public service announcement of media than rural consumers, and the publicity effect will also affect the formation of safety trust to a certain extent. Rural consumers are greatly influenced by the word-of-mouth of relatives and friends. Therefore, the consumer’s residential area plays a regulating role in the formation process of reputation information influencing the safety trust in CIMP. *Education level*. Education level will affect consumers’ attitudes towards food safety information [52,53]. With more and more word-of-mouth information collected by consumers with higher level of education, they will seriously consider and compare the safety information transmitted by media or relatives and friends, and will be more able to resist online rumors and negative word-of-mouth. Therefore, education level plays a regulating role in the formation process of reputation information influencing the safety trust in CIMP [15,43,45]. To this end, this paper puts forward the following assumptions:

**Hypothesis** **(H2e).** *Consumer involvement, emotional system, cognitive system and other consumer psychological characteristics plays a regulating role in the formation process of reputation information influencing the safety trust in CIMP.*

**Figure 2:**
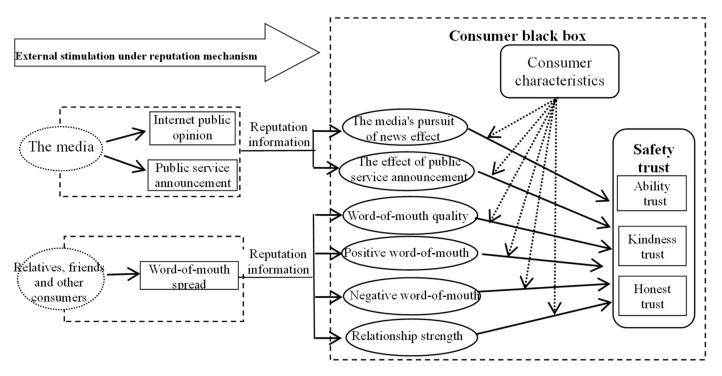
An analysis framework of the influence mechanism of reputation information on the formation of safety trust in CIMP.

**Hypothesis** **(H2f).** *Consumer’s residential area, level of education and other consumer demographic characteristics plays a regulating role in the formation process of reputation information influencing the safety trust in CIMP (Figure 2).*

## 3. Questionnaire Design and Data Statistics

### 3.1. Questionnaire Design

The first part of the questionnaire for this paper is personal information such as the consumer’s current residential area, level of education and the consumer behavior information of infant milk powder. The latter three parts are the trust rank measurement index system scale for the CIMP safety, the consumer characteristic scale and the consumer psychological perception variable scale for reputation information of infant milk powder. All of these parts are completed by the Likert scale 5-point method [54]. Respondents were asked to rate the relevant questions according to the true degree of trust or consent of their own. The following section mainly introduces consumer psychological perception variable scale for reputation information of infant milk powder, as shown in Table 1:

(1) The variable of the media’s pursuit of news effect (NE): the media for its own economic interests, often pursue news effect, tend to report often negative, a sensation effect of food safety incidents, or exaggerated, injustice will be one-sided reports of safety information, through the “hype” way to attract the attention of consumers, it will no doubt make consumers more doubt to food safety [55]. 

(2) The variable of the effect of public service announcement (PW): the food safety law also emphasizes the role of news media in public welfare publicity and supervision by public opinion, and media public welfare publicity should be an important way for consumers to obtain domestic infant milk powder safety knowledge. 

(3) The variable of word-of-mouth quality (MQ): Bhattacher et al. proposed that word-of-mouth quality refers to the degree of persuasion that consumers can feel from the word-of-mouth information they receive [39]. Whether the content of word-of-mouth information is easy to understand and whether it describes the reviewer’s experience of the product or service in detail will affect the perception of the credibility of the comment [56]. This study will reflect the public praise quality of domestic infant milk powder through the details, professionalism and information value of domestic infant milk powder transmitted by relatives and friends. 

(4) The variable of positive word-of-mouth (PM): positive word of mouth often expresses consumers’ satisfied consumption feelings and praises the products or services provided by enterprises. 

(5) The variable of negative word-of-mouth (NM): negative word of mouth is to express consumers’ dissatisfaction and point out product defects or service deficiencies of the enterprise. In this study, the positive/negative public praise of domestic infant milk powder was reflected by consumers’ perceived positive or negative status of their relatives and friends on the safety of domestic infant milk powder. 

(6) The variable of relationship strength (TS): the Frenzen scale was used to measure closeness, intimacy, support and other aspects, such as consumers’ perceived similarity with the information sender, familiarity, willingness to offer help, and ability to discuss personal issues. Wang used the scale for reference to measure variables [57]. In this study, the relationship strength was reflected by the perceived closeness between consumers and their relatives and friends, whether they could take the initiative to discuss the safety issues of domestic infant milk powder, and whether they would be persuaded by the suggestions of relatives and friends.

### 3.2. Data Statistics

The research objects selected in this paper are divided into three types: potential customers- women ready for pregnancy and in pregnancy, current customers-parents who need to buy infant milk powder, and lost customers-parents whose need to buy infant milk powder is just ended and whose babies are not more than 4 years old. Based on the investigation of urban areas, county towns, villages and towns and rural areas in six provinces (cities) of Guangdong, Beijing, Heilongjiang, Hebei, Sichuan and Gansu, 106, 125, 134, 140, 119 and 115 questionnaires were collected respectively, with 739 in total, 685 in effect, and an effective rate of 92.69%.

Statistics of the 685 respondents show the following facts: The majority of respondents are female, accounting for 68.76%, which is related to the fact that the infant milk powder market is dominated by female consumers. Respondents currently living in urban and county towns account for 59.42%. Respondents aged 21–40 account for 88.61%, indicating that the buyers of infant milk powder are basically the parents of the babies. Respondents received college or university undergraduate education account for 66.72%. Since the buyers of this group have a higher level of education, they have strong ability to collect information and make rational analysis when making trust decisions. The monthly income of the respondents’ families is mostly in two stages: 6001~8000 Yuan and 8001~10,000 yuan. Judging from the number of children in the respondents’ families, there is still only one child in more than 60% of the families, although the two-child policy has been fully carried out. The age of actual consumers of infant milk powder in the respondents’ families is mainly between 1 and 3 years old, accounting for 35.33%. Some of these babies have been consuming milk powder since birth, and the others have changed from being breast-fed to consuming milk powder. Respondents who purchase CIMP account for 47.88%, and most of the residents in towns and villages chose to buy CIMP. The main channels for respondents to buy infant milk powder are maternal and child supplies specialty store, online shopping platforms and business super.

## 4. Empirical Analysis

### 4.1. Reliability and Validity Analysis

The purpose of reliability test is to measure the consistency or stability of each scale in the relational model. Cronbach’s alpha coefficient analysis is used to test reliability commonly, and when the value of the system is greater than 0.7, it indicates that the latent variable scale has a high reliability. When the number of test questions corresponding to a latent variable is less than 6, the coefficient value greater than 0.6 indicates that the latent variable scale has a high reliability [58].In this paper, SPSS 23.0 software (IBM, Armonk, N.Y., USA) is used to test the reliability of all the latent variable indexes of “the influence mechanism of reputation information on the formation of safety trust in CIMP”. The Cronbach’s alpha obtained is all above 0.8, which indicates that the measurement scale of the influence mechanism of reputation information on the formation of safety trust in CIMP has passed the reliability test and its internal consistency is good (Table 2).

Validity tests are used to measure the accuracy of each scale reflecting latent variable characteristics, of which content validity test and structural validity test are often used. In the scale designed in this paper, latent variables and test topics refer to the research literature of experts in relevant fields at home and abroad, and some latent variables and test topics are repeatedly used by different experts. In addition, in the research process of this paper, a lot of discussion and communication was held with the teachers and students of the research group for the design of this scale. The revised and improved scale has been unanimously approved, so it can be concluded that the content validity of the scale is good. Structural validity is mainly about confirmatory factor analysis, which is decided by KMO and Bartlett’s spherical test. After calculation, values of the former are all above 0.7, which meets Kaiser’s minimum standard of more than 0.5. The significance probability value of the latter is close to 0, rejecting the null hypothesis that the correlation coefficient matrix is identity matrix. And the factor loading is not abnormal, indicating that the structural validity of the scale is good.

### 4.2. Hypothesis Testing

The fitting degree of the structural equation model of the influence mechanism of reputation information on the formation of safety trust in CIMP is shown in Table 3. By comparing the evaluation index values with the standard values, it is found that all the index values conform to the evaluation standard, which shows that the structural equation model designed in this paper has a good fit with the sample data.

With Amos 21.0 (IBM, Armonk, N.Y., USA) to test the research hypothesis in this paper, the results show that all the other five factors have a significant impact on safety trust, while only the public service announcement effect of the media has no significant impact on safety trust. Among them, negative word-of-mouth is the most influential, followed by word-of-mouth quality, media pursuit degree of “news effect”, relationship strength and positive word-of-mouth successively. The degree of media pursuit of “news effect” has a significant negative impact on the formation of safety trust in CIMP. H1a passed the significance test at 1%. As the media often focus on economic benefits when choosing what to report, they often report information with strong news effects. This easily leads to distortion of relevant reports and reputation commentary information, and cannot really exert the value of information in the communication process. Moreover, the phenomenon of online hype is often reported, which increases the prejudice of consumers against media reports. All these make consumers question the authenticity, integrity and accuracy of the reputation information of infant milk powder. Consumers’ perception of the media’s pursuit of “news effect” will especially enhance consumers’ distrust. The effect of the public service announcement of the media has a positive impact on safety trust, but not at a significant level, which indicates that the public service announcement of the media cannot significantly promote the formation of safety trust in CIMP. H1b failed the test. The reason for this result may be because that the media do not give enough promotion to the safety reputation information of CIMP, and most consumers’ perception of the public service announcement of the media is not sufficient. The word-of-mouth quality of relatives and friends has a significant positive impact on the formation of safety trust in CIMP. H2a passed the significance test at 1%. High-quality word-of-mouth information is more convincing, which can help consumers form a clear understanding of the safety reputation of CIMP, make consumers feel that word-of-mouth information is effective and useful, enhance consumers’ acceptance of the content of reputation information, and promote the formation of consumers’ trust. This is consistent with the views of Cheung and other scholars [59]. (Positive word-of-mouth has a significant positive effect on the formation of safety trust in CIMP. H2b passed the significance test at 5%. The more positive word-of-mouth consumers receive, the more likely they are to accept the safety of CIMP, and the more helpful it is for the formation of consumer trust. Negative word-of-mouth has a significant negative effect on the formation of safety trust in CIMP, and its influence degree is stronger than positive word-of-mouth. Due to the safety sensitivity of infant milk powder, negative word-of-mouth is easier to attract more attention. This is the reason why negative word-of-mouth has a greater impact than positive word-of-mouth, which plays a strong inhibitory role on the formation of consumer trust. H2c passed the significance test at 1%. This is consistent with the views of Arndt J, Chevalier J.A. and other scholars [33,41]. (6) The relationship strength has a significant positive effect on the formation of safety trust in CIMP. H2d passed the significance test at 1%. When relatives and friends transmit reputation information of infant milk powder to consumers, different reputation recommendation effects will be produced due to different relationship strength. The higher the relationship strength is, the deeper the consumer’s acceptance of reputation information is, and the more it can promote the formation of consumer trust (Table 4).

### 4.3. Empirical Test and Result Analysis on the Regulating Effect of Consumer Characteristics

Multi-group structural equation analysis is often used to test regulatory variables in consumer behavior research. In this paper, Amos21.0 was used for multi-group analysis of consumer characteristics. Five hypothetical models are set up, including unrestricted model, covariance equal model, variance equal model, path coefficient equal model and model invariance model. By means of comparative analysis of the fit measure, path coefficient equal model is finally selected as the best model for multi-group analysis with consumer involvement as the regulating variable, covariance equal model with emotional and cognitive system, path coefficient equal model with urban and rural areas, and unrestricted model with education level. The results show that, in H2a: ST←MQ, consumers’ perceptions of word-of-mouth quality in the highly involved group, rational group, urban group and high-educated group have a stronger influence on the formation of safety trust. The possible reason is that, in the process of gradually forming trust among consumers, these consumers are, the more information they collect, the more attention they pay to the deeper information and the greater their demand. The better quality of the word of mouth of relatives and friends can attract more attention from consumers. While in H1a: ST←NE, H2b: ST←PM, H2c: ST←NM and H2d: ST←TS, consumers’ perceptions of media pursuit of “news effect”, positive word-of-mouth, negative word-of-mouth and relationship strength in low involvement group, perceptual group, rural group and low-educated group have stronger influence on the formation of safety trust (Table 5).

## 5. Conclusions

The purpose of this study is to explore the influence mechanism of reputation information on the formation of safety trust in CIMP in the context of dairy industry revitalization. Of the process of the formation of safety trust in CIMP, the reputation information source, the transmission path and the influence mode of information, as well as the influence mode of consumer characteristics (including consumer involvement, emotion or cognition, urban or rural areas, and level of education) are analyzed in detail.

The research results show the following facts: In the reputation information sources, the reputation information transmitted by relatives and friends plays a stronger role in the formation of safety trust in CIMP than the media. The media’s pursuit of “news effect” and negative word-of-mouth have a significant negative impact on the formation of safety trust in CIMP, and negative word-of-mouth has a greater impact on the formation of safety trust in CIMP. Word-of-mouth quality, positive word-of-mouth and relationship strength have significant positive effects on the formation of safety trust in CIMP, and the degree order from strong to weak to promote the formation of safety trust in CIMP is word-of-mouth quality, relationship strength and positive word-of-mouth. We also found that consumer characteristics play a regulating role in the formation of safety trust in CIMP. The quality of word-of-mouth perceived by consumers from highly involvement group, rational group, urban group and high-educated group has a stronger influence on the formation of safety trust. The degree of media pursuit of “news effect”, positive word-of-mouth, negative word-of-mouth and relationship strength perceived by consumers from low involvement group, emotional group, rural group and low-educated group have stronger influence on the formation of safety trust.

Based on the above results, the following suggestions are made in this research. First, a network public opinion monitoring system and reputation crisis early warning mechanism of CIMP safety, which is jointly organized by the government, media and infant milk powder enterprises, shall be established. And the combination of manual monitoring and internet technology monitoring shall be applied to obtain the dynamic reputation information of network public opinion of CIMP safety. Second, the media establish a popular science service network platform for infant milk powder and a market supervision platform. The government releases timely the information of the safe production of CIMP enterprises and the safety of milk powder on the official website. Third, under the supervision of the government, the safety trust status of consumers in all aspects of cultivation, processing and sales should be evaluated in a timely manner by combining the self-evaluation by the infant milk powder industry and enterprises with the evaluation by a third-party professional organization, and an early warning system for safety trust in CIMP and corresponding response plans should be established. Fourth, CIMP enterprises should establish a milk powder traceability system and pay regular return visits to buyers so that regular customers can become loyal consumers. Offline promotion and public relations activities for infant milk powder should be held in order to strengthen the word-of-mouth marketing of CIMP safety and better guide public opinion.

## Figures and Tables

**Table 1:** Psychological perception scale based on reputation information.

Latent Variable	Number	Test Topic
The media’s pursuit of news effect (NE)	NE1	The media exaggerate the safety accident of Chinese milk powder for economic benefit.
	NE2	The media distort or unilaterally report the safety accident of Chinese milk powder for economic benefits.
	NE3	The media tend to produce news effects through negative reports.
The effect of public service announcement (PW)	PW1	Public service announcement in the media will let me learn a lot about the safety of infant milk powder.
	PW2	Public service announcement in the media will affect my choice of CIMP.
	PW3	Public service announcement in the media promotes the popularization of safety knowledge of CIMP.
Word-of-mouth quality (MQ)	MQ1	Some relatives and friends tell me about the function or eating experience of CIMP in more detail.
	MQ2	The relatives and friends who give me advice have rich safety knowledge or eating experience of CIMP.
	MQ3	Relatives and friends give me a lot of valuable safety information of CIMP.
Positive word-of-mouth (PM)	PM1	The vast majority of relatives and friends believe that CIMP is safe.
	PM2	The vast majority of relatives and friends believe that CIMP is safer than imported milk powder.
	PM3	Relatives and friends often recommend CIMP to me.
Negative word-of-mouth (NM)	NM1	Relatives and friends generally question the safety of CIMP.
	NM2	Relatives and friends who buy CIMP complain to me about the safety of it.
	NM3	Most of my relatives and friends recommend import infant milk powder to me.
Relationship strength (TS)	TS1	The relatives and friends who give me advice are very close to me.
	TS2	I will discuss matters about infant milk powder with relatives and friends who give me advice.
	TS3	I will change my mind of buying Chinese or imported infant milk powder on the advice of my relatives and friends.

**Table 2:** Consistency coefficient of each variable.

Latent Variable	Number of Items Tested	Cronbach’s Alpha	Latent Variable	Number of Items Tested	Cronbach’s Alpha
The media’s pursuit degree of “news effect”	3	0.837	Safety trust	——	——
The effect of public service announcement	3	0.839	Ability trust	4	0.888
Word-of-mouth quality	3	0.880	Kindness trust	5	0.928
Positive word-of-mouth	3	0.815	Emotional trust	4	0.879
Negative word-of-mouth	3	0.829			
Relationship strength	3	0.813			

Source of data: according to SPSS output results.

**Table 3:** The fitting degree of the structural equation model of the influence mechanism of reputation information on the formation of safety trust in CIMP.

Evaluation Norm.	*χ*^2^/df	RMSEA	GFI	NFI	CFI	PNFI	PGFI
Evaluation standard	<5	<0.08	>0.9	>0.9	>0.9	>0.5	>0.5
Index value	2.202	0.042	0.926	0.934	0.963	0.824	0.765

Source of data: collated according to Amos output results.

**Table 4:** Relevant research hypothesis test results on the influence mechanism of reputation information on the formation of safety trust in CIMP.

Path	Estimate	C.R.	*p*	Conclusion
H1a: ST←NE	−0.214	−3.950	***	Support
H1b: ST←PW	0.059	1.728	0.084	Nonsupport
H2a: ST←MQ	0.243	5.046	***	Support
H2b: ST←PM	0.147	2.908	**	Support
H2c: ST←NM	−0.317	−6.103	***	Support
H2d: ST←TS	0.162	3.286	***	Support

Notes: ** means *p* < 0.01, *** means *p* < 0.001. The path coefficient in the table is the normalized regression coefficient. C.R. is the critical ratio of non-standardized regression coefficients. Path H2a-H3d is consistent with previous assumptions.

**Table 5:** The estimation results of multi-group analysis of adjustment variables.

Path	With “Psychological Characteristics of Consumers in Processing Information” as the Regulating Variable				With “Consumer Demographic Characteristics” as the Regulating Variable			
	According to Consumer Involvement		According to Emotional/Cognitive System		According to Urban/Rural Area		According to Level of Education	
	High Involvement Group (*n* = 382)	Low Involvement Group (*n* = 303)	Emotional Group (*n* = 365)	Rational Group (*n* = 320)	Urban Group (*n* = 407)	Rural Group (*n* = 278)	High-Educated Group (*n* = 377)	Low-Educated Group (*n* = 308)
H1a:ST←NE	−0.178 *	−0.264 ***	−0.217 **	−0.216 **	−0.179 *	−0.261 ***	−0.193 *	−0.251 ***
H1b:ST←PW	0.062	0.058	0.045	0.087	0.069	0.055	0.063	0.061
H2a:ST←MQ	0.285 ***	0.160 *	0.188 **	0.288 ***	0.284 ***	0.154 *	0.281 ***	0.167 *
H2b:ST←PM	0.148 *	0.172 *	0.154 *	0.146 *	0.155 *	0.166 *	0.135 *	0.174 *
H2c:ST←NM	−0.305 ***	−0.326 ***	−0.336 ***	−0.301 ***	−0.312 ***	−0.318 ***	−0.305 ***	−0.325 ***
H2d:ST←TS	0.157 *	0.176 *	0.216 **	0.109 *	0.146 *	0.193 *	0.151 *	0.187 *

Note: * means *p* < 0.05, ** means *p* < 0.01, *** means *p* < 0.001. The path coefficient in the table is the normalized regression coefficient. Paths H2a-H3d are consistent with the previous assumption.
